# Clinical Utility of Disease Activity Indices in Predicting Short-Term Response to Biologics in Patients with Ulcerative Colitis

**DOI:** 10.3390/jcm13123455

**Published:** 2024-06-13

**Authors:** Filip Romaniuk, Anna Franus, Aleksandra Sobolewska-Włodarczyk, Anita Gąsiorowska

**Affiliations:** Department of Gastroenterology, Medical University of Łódź, 90-419 Łódź, Poland; anna.franus@stud.umed.lodz.pl (A.F.); aleksandra.sobolewska-wlodarczyk@umed.lodz.pl (A.S.-W.); anita.gasiorowska@umed.lodz.pl (A.G.)

**Keywords:** ulcerative colitis, vedolizumab, infliximab, UCEIS, Mayo Score, remission, remission induction

## Abstract

**Background**: The Mayo Score [MS], endoscopic Mayo Score [eMS] and the Ulcerative Colitis Index of Severity [UCEIS] are employed in the assessment of ulcerative colitis [UC] severity. This study compared the aforementioned indices in terms of predictory value for response to remission induction treatment with anti-TNF and anti-integrin biologics. **Methods**: A total of 38 patients were retrospectively evaluated in the study, 23 male and 15 female, aged 18–74 years old who had undergone a total of 53 biological therapy courses with either infliximab [IFX] or vedolizumab [VDZ] at the Department of Gastroenterology of the Medical University of Łódź. The clinical and endoscopic activity of UC was assessed at the outset of biological therapy and the 14th week remission induction assessment juncture. **Results**: The study analyzed 19 IFX and 34 VDZ treatment courses. The response rate of patients receiving IFX reached 73.67% and the response rate was 58.82% for VDZ. The mean MS, eMS and UCEIS improved among all patient groups: 8.316 ± 1.974 to 4.158 ± 2.218 (*p* < 0.05), 2.632 ± 0.597 to 1.790 ± 0.713 (*p* < 0.05) and 4.790 ± 1.745 to 3.000 ± 1.453 (*p* < 0.05) for IFX, 7.088 ± 2.234 to 3.618 ± 2.412 (*p* < 0.05), 2.706 ± 0.524 to 1.677 ± 1.065 (*p* < 0.05) and 4.235 ± 1.350 to 2.735 ± 1.880 (*p* < 0.05) for VDZ. **Conclusions**: The outcome assessment in induction treatment of UC includes clinical data and endoscopic evaluation. Severity of inflammatory lesion activity according to the eMS and UCEIS indices correlates with the overall disease presentation as evaluated with MS. The UCEIS provides an overall better predictor for biological induction treatment when compared with the eMS in both patient groups, particularly in those receiving VDZ. It provides a promising alternative to the eMS and can be employed for both initial disease severity assessment as well as for treatment response monitoring.

## 1. Introduction

Ulcerative colitis [UC] is a chronic inflammatory condition of unknown etiology affecting the colon and rectum characterized by ulceration of the colonic mucous membrane [1]. Common symptoms include abdominal pain, hematochezia and weight loss. Associated extraintestinal symptoms encompass inflammatory arthropathies and primary sclerosing cholangitis [PSC] [2,3].

Typically, inflammation spreads proximally from the rectum towards the ileocecal valve. The clinical presentation is varied, with some patients presenting with inflammation contained only to the rectum while others suffer from complete inflammation of the entire colonic mucosa, known as pancolitis [4]. Furthermore, UC is characterized by recurring periods of exacerbation and remission [1]. Diagnosis is mainly based on endoscopy, histopathological examination and clinical presentation [5].

Treatment options are varied and depend on the severity of clinical presentation with the therapeutic goal usually being clinical and endoscopic corticosteroid free remission [6]. Management of UC is usually divided into remission induction and remission maintenance.

Mild to moderate cases are usually treated with 5-aminosalicylic acid [5-ASA] or its derivatives with the addition of corticosteroids for those who do not achieve remission with 5-ASA on their own.

Treatment of moderate to severe UC typically begins with oral or intravenous corticosteroids followed by 5-ASA, drugs from the thiopurine family followed by biological drugs such as infliximab [IFX] and vedolizumab [VDZ]. Extreme, unresponsive to treatment cases may require surgical intervention [7].

There is strong scientific evidence of the efficacy of monoclonal antibodies in the treatment of patients with UC [8,9,10]. Such agents include IFX and VDZ.

IFX is an anti-tumor necrosis alpha [anti-TNF-α] chimeric human-murine IgG1 monoclonal antibody produced in Chinese hamster ovary [CHO] cells by recombinant DNA technology used in the treatment of UC, Crohn’s disease [CD] and several rheumatological conditions. It has been approved for both induction and maintenance of remission in UC. It works by inhibiting TNF-α, a cytokine with a broad spectrum of pro-inflammatory activity.

VDZ is a humanized IgG1 monoclonal antibody produced in CHO cells by recombinant DNA technology that binds to the α4β7 integrin, resulting in gut specific anti-inflammatory activity.

The expression of α4β7 on lymphocytes allows for binding with the mucosal addressin cell adhesion molecule [MAdCAM]. This mechanism is responsible for T-cell migration into gut lymphoid tissues [11], as such anti α4β7 agents result in diminished inflammatory activity resulting in a reduction of UC symptoms [12].

As there presently exists no curative treatment for UC, patients are left with no choice but to commit to lifelong therapy. Identifying prognostic factors that would allow us to predict a given treatment regimen’s outcome could shorten hospitalizations, improve patients’ quality of life and minimize time spent on otherwise futile treatment options.

This study aims to provide a comparison between the Ulcerative Colitis Endoscopic Index of Severity [UCEIS], the Mayo Score [MS] and the Partial Endoscopic Mayo Score [eMS] in patients receiving IFX or VDZ for the treatment of UC. The basis for such comparison will be the measurement of both the clinical and endoscopic response to treatment at the remission induction assessment point after 14 weeks of therapy.

## 2. Materials and Methods

### 2.1. Patients

This retrospective clinical study was performed in 38 adult patients, 18–74 years of age, of Caucasian origin with UC who had undergone therapy with either IFX or VDZ—two main biologics used at our department—up until at least the 14th week of treatment. Our study analyzed 19 individual courses of IFX and 34 individual courses of VDZ therapy performed at the Department of Gastroenterology of the Medical University of Łódź.

UC was diagnosed and confirmed according to clinical, endoscopic, radiological and histological guidelines published by the European Crohn’s and Colitis Organization [ECCO].

Patients were evaluated according to the MS, the eMS and the UCEIS, both at the initiation of treatment and at the 14th week remission induction assessment point.

The MS is a complex scoring system based on four segments: overall physician assessment, stool frequency, rectal bleeding and endoscopy. Each segment is graded from 0 to 3, giving a score of 0 to 12. A score of 3 to 5 points indicates mildly active disease, a score of 6 to 10 points indicates moderately active disease and a score of 11 or 12 indicates severely active disease.

The UCEIS is a scoring system based on three segments: vascular pattern, bleeding, erosions and ulcers. The vascular pattern segment is graded from 0 to 2, while the bleeding and erosion or ulcers are both graded between 0 to 3. A minimum score of 0 indicates no disease activity and a maximum score of 8 indicates severe disease.

The enrolment criteria for IFX and VDZ therapy included clinically significant UC exacerbation, failure or intolerance of earlier therapies such as 5-ASA and its derivatives, immunosuppressants such as azathioprine and its derivatives or corticosteroids. All patients qualified for this study had luminal activity of UC, examined via endoscopy prior to biological therapy.

Of 44 patients who had undergone a total of 70 courses of therapy (22 of IFX and 48 of VDZ), 17 courses were excluded from the study. Exclusion criteria included incomplete data regarding physical assessment and endoscopy reports (nine courses), change of administered drug (three courses), discontinuation of therapy due to anaphylaxis (one course), unknown reasons (three courses) and patient death (one course).

Patients included in the study received either 5 mg per kg of body mass IFX or 300 mg iv. VDZ solution.

Response to either IFX or VDZ therapy was defined as a decrease of at least three total Mayo Score points at week 14.

A clinical assessment of all enrolled patients was performed at each visit related to biological drug administration. Based on these medical examinations, according to the National Drug Program [NDP] and local Summary of Product Characteristics, UC patients with hyperreactivity to either VDZ or IFX, a precancerous condition or malignancy diagnosed within 5 years of study enrolment, chronic heart, kidney, liver or respiratory failure, demyelinating disease, severe active or opportunistic infections (e.g., progressive multifocal leukoencephalopathy), pregnancy or during breastfeeding were excluded. At inclusion in the study (before the first administration of either IFX or VDZ), patients had their blood serum C-reactive protein [CRP], hemoglobin [Hgb], leukocyte [WBC], lymphocyte [LYM] and calprotectin [CAL] levels assessed. Their pre-treatment reliance on oral corticosteroids and earlier exposure to biological treatment was also noted. Patients who had earlier exposure to any kind of biological therapy (especially one based on monoclonal antibodies) are defined as biopositive while those who had not are defined as bionaïve.

### 2.2. Collection of Blood Samples and Blood Analysis

An adequate amount of venous blood was taken from all patients into standardized tubes containing ethylenediaminetetraacetic acid [EDTA] to determine CRP, Hgb, WBC and LYM serum levels. All samples for analysis were obtained before the first IFX/VDZ administration. Blood analysis was performed within 2 h after collection.The adult normal reference range for Hgb is between 13 and 18 g/dl for men and between 12 and 16 g/dl for women, respectively, between 4000 and 10,000/µL for WBC, between 1000 and 4000/µL for LYM, <5 mg/L for CRP and <50 µg/g for CAL.

### 2.3. Statistical Analysis

The analysis of data collected in the study was performed with the statistical package Statistica 13.1 (StatSoft, Inc., Tulsa, OK, USA). The analyzed results are presented as a mean standard deviation regarding continuous variables and as numbers and percentages referring to categorical variables. The estimation of the normality of the distribution of the examined quantitative parameters was executed with the Shapiro–Wilk test. Data was assessed with the Student’s *t* test, the non-parametric Mann–Whitney U test and the Sign test when comparing pre- and post-treatment endoscopic scale scores.

Categorical variables were compared using Fischer’s exact test. Correlations were tested using Spearman’s rank correlation coefficient.

The receiver operating characteristic [ROC] curve analysis was also performed to evaluate the predictive value of endoscopic scores in response to biological treatment.

In all the analyses, the probability value of *p* < 0.05 was considered statistically significant.

## 3. Results

**Figure 1:**
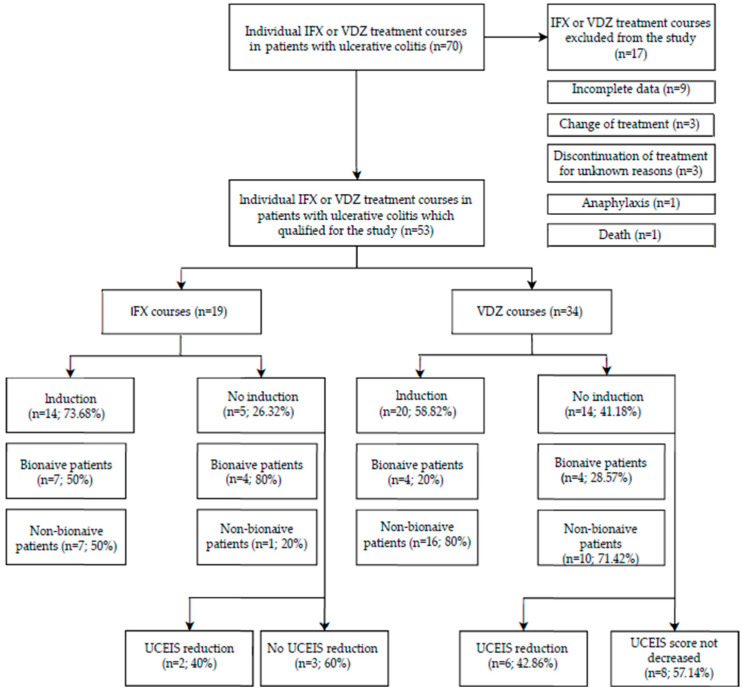
Baseline characteristics of patients included in the study.

A total sample of 37 patients who had undergone either IFX or VDZ therapy until the 14th week induction therapy mark were enrolled in our study: 22 men (59.46%) and 15 (40.54%) women. Individual treatment courses equated to 19 IFX (38.2%) and 34 VDZ (61.8%) for a total of 53. Due to the number of courses assessed being greater than the number of individual patients admitted to the study, each course of treatment with either IFX or VDZ was considered separately. Baseline characteristics of patients included in the study are shown in Figure 1.

The most common localization of inflammatory lesions was pancolitis (*n* = 32), left colon (*n* = 17) and rectum (*n* = 4). The mean duration of the disease was 7.849 ± 5.731 years. The average patient age at the beginning of treatment was 37.96 ± 13.282 years.

Among the patients who had received VDZ, 85.29% (*n* = 29) relied on prior glucocorticoid therapy compared with 94.74% (*n* = 18) of the recipients of IFX. A total of 29.41% (*n* = 10) of the 34 assessed VDZ courses were performed in bionaïve patients while 57.89% (*n* = 11) of the 19 assessed IFX courses were performed in bionaïve patients.

Mean trough levels of CRP amounted to 11.19 mg/L ± 17.8299 in VDZ patients and 22.56 mg/L ± 34.5233 for IFX at the first dose. Mean trough levels of Hgb for male patients receiving VDZ amounted to 14.30 g/dL ± 1.283 and this amounted to 12.86 g/dL ± 1.16 for female patients. Mean trough levels of Hgb for male patients receiving IFX amounted to 12.13 g/dL ± 2.114, and these were 12.73 g/dL ± 1.366 for female patients. WBC in VDZ patients amounted to an average of 8.672/µL ± 2.974, and WBC in IFX patients amounted to an average of 9.873/µL ± 2.983. LYM in VDZ patients amounted to an average of 2.079/µL ± 1.104, while LYM in IFX patients amounted to an average of 1.942/µL ± 0.988. CAL in VDZ patients amounted to an average of 1174.06 µg/L ± 761.84, while CAL in IFX patients amounted to an average of 947.91 µg/L ± 846.20. Baseline characteristics of the IFX patient sample are reported in Table 1.

There was no significant difference between the IFX receiving responder and non-responder groups regarding sex (*p* = 0.6641), age (*p* = 0.3371), location of inflammatory lesions (*p* = 0.4097), prior exposure to biological drugs (*p* = 0.2668), prior glucocorticoid use (*p* = 0.7368), disease duration (*p* = 0.7311), hemoglobin (*p* = 0.8644), white blood count (*p* = 0.7864), lymphocyte count (*p* = 0.5875), CRP (*p* = 0.5335) and calprotectin (*p* = 0.6098). Baseline characteristics of the VDZ patient sample are reported in Table 2.

There was no significant difference between the VDZ receiving responder and non-responder groups regarding sex (*p* = 0.1194), age (*p* = 0.9860), location of inflammatory lesions (*p* = 1.000), prior glucocorticoid use (*p* = 0.3650), disease duration (*p* = 0.9125), hemoglobin (*p* = 0.5785), white blood cell (*p* = 1.000), lymphocyte count (*p* = 1.000), CRP (*p* = 0.6358) and calprotectin (*p* = 0.0758).

Prior exposure to biological drugs presented itself as the only outlier (*p* = 0.0204), with the response group being significantly skewed towards bionaïve patients (70% vs. 30%).

### 3.1. Short-Term Response

In both groups of patients, the Sign test was used to assess pre- and post-treatment values.

Among the assessed IFX courses, 14 (73.68%) responded to treatment with statistically significant reductions of UCEIS/eMS/MS. Mean UCEIS, MS and eMS scores in IFX patients are shown in Table 3.

Of the 14, 7 were biopositive and 7 were bionaïve, both also achieved statistically significant reductions (*p* = 0.0412, *p* = 0.0412, *p* = 0.0233 and *p* = 0.0233, *p* = 0.0233, *p* = 0.0233).

Of the five non-responders, four (80%) were bionaïve and one was biopositive (20%). Such a sample of patients in this group is too small to draw any concrete conclusions.

Of the 34 VDZ courses, 20 (58.82%) responded to treatment, while 14 (41.18%) did not. Of the 20 responders, only 6 (30%) were bionaïve, while 14 (70%) were biopositive. Mean UCEIS, MS and eMS scores in VDZ patients are shown in Table 4.

All of the 20 achieved a statistically significant reduction in mean UCEIS/eMS/MS scores, and so did the 14 biopositives when considered separately (*p* = 0.00937, *p* = 0.00257, *p* = 0.001496).

In the six bionaïves only the MS managed to produce a narrowly significant result (*p* = 0.0736, *p* = 0.0736, *p* = 0.04123).

Of the 14 non-responders, 4 (28.57%) were bionaïve, while 10 (71.43%) were biopositive.

Among the 10 biopositive non-responders, only the MS managed to produce a significant difference between pre- and post-treatment values (*p* = 0.4497, *p* = 0.3711, *p* = 0.00766).

Among the remaining four bionaïve non-responders, there was no significant reduction in either of the assessed scores.

Surprisingly, the 14 non-responders assessed according to the MS achieved a significant improvement despite not fulfilling NDP criteria for remission.

### 3.2. Correlations

Spearman correlation coefficient analysis was performed in both groups of patients.

#### 3.2.1. IFX

A positive correlation between pre-treatment values of MS-UCEIS r(17) = 0.49 (*p* = 0.0327) and a strong correlation between eMS-UCEIS r(17) = 0.681 (*p* = 0.001315) was observed.

No correlation between pre-treatment values of MS-eMS r(17) = 0.2 (*p* = 0.411) was noted.

Strong positive correlations were observed in all post-treatment comparisons, r(17) = 0.688 (*p* = 0.00113) for MS-UCEIS, r(17) = 0.746 (*p* < 0.001) for MS-eMS and r(17) = 0.943 (*p* < 0.001) for eMS-UCEIS.

#### 3.2.2. VDZ

No significant correlation between pre-treatment values of MS-UCEIS r(32) = 0.2566, (*p* = 0.15633) was found. A better positive correlation between MS-eMS r(32) = 0.426, (*p* = 0.0172) and a strong correlation between pre-treatment values of eMS-UCEIS r(32) = 0.725, (*p* < 0.001) was observed.

Very strong post-treatment positive correlations were observed among all groups. A value of r(32) = 0.73329 (*p* < 0.001) for MS-UCEIS, r(32) = 0.725 (*p* < 0.001) MS-eMS and r(32) = 0.8259 (*p* < 0.001) for eMS-UCEIS was observed.

### 3.3. ROC Curve Analysis

The receiver operating characteristic curve [ROC] analysis was used to evaluate the ability of UCEIS, MS and eMS to predict remission induction in patients with ulcerative colitis. Optimal cut-off values were devised. Analyses with both sensitivity and specificity values of at least ≥50% were considered significant. If more than one analysis was applicable, the one with better sensitivity and specificity was included.

**Figure 2:**
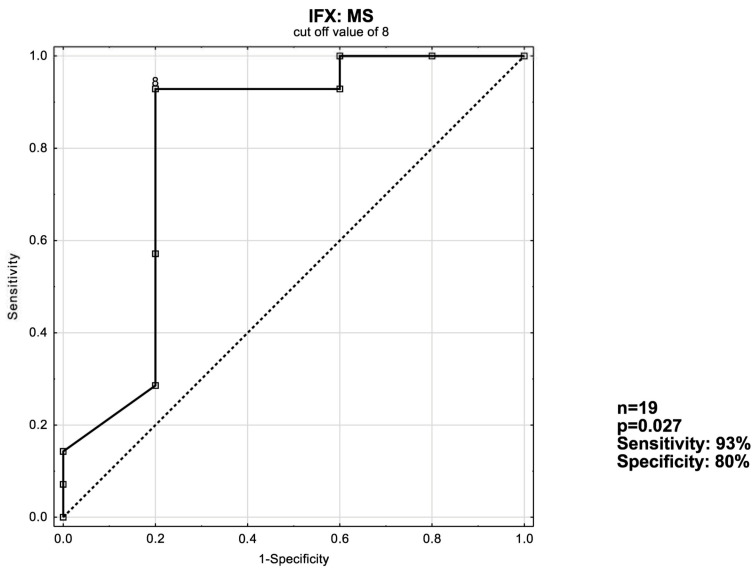
ROC curve for short-term response in the IFX: MS patient group.

**Figure 3:**
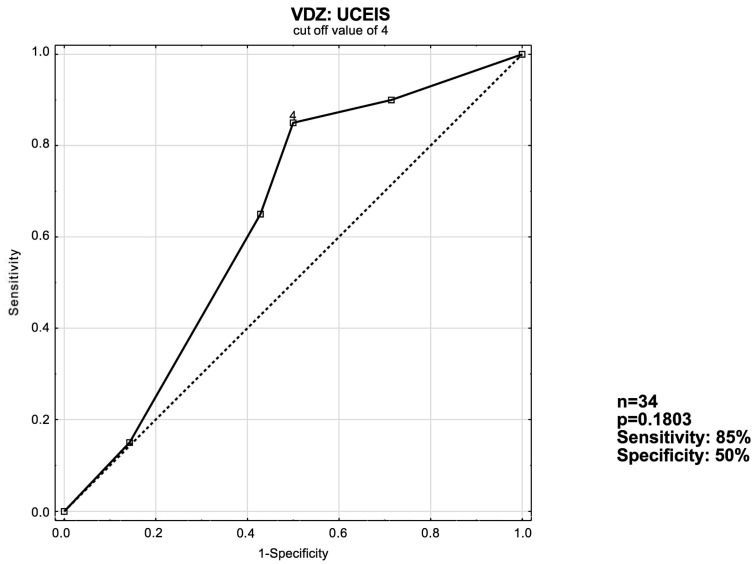
ROC curve for short-term response in the VDZ: UCEIS patient group.

As shown in Figure 2, a cut-off value of MS ≥ 8 with an area under the ROC curve [AUC] of 0.814 is the ideal value for predicting short-term response in IFX patients. In VDZ patients, the optimal cut-off value was UCEIS ≥ 4 with an AUC of 0.638 (Figure 3)—along with a suboptimal *p*-value of ~0.18.

**Figure 4:**
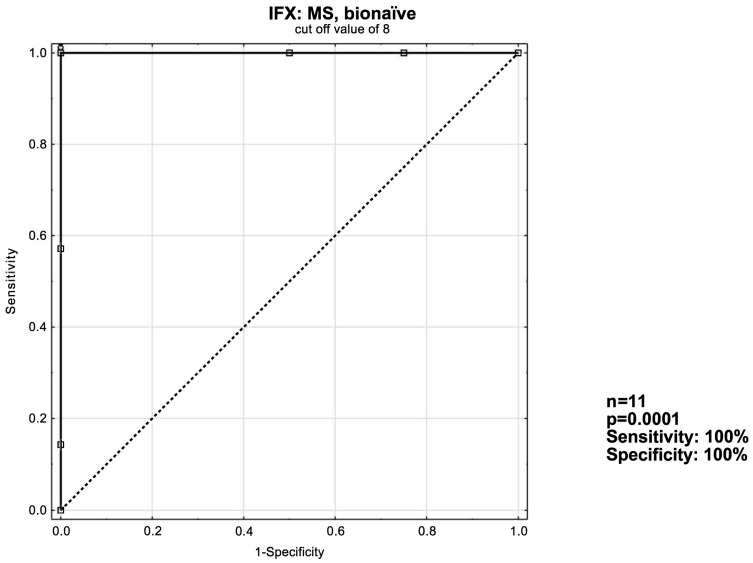
ROC curve for short-term response in the IFX: MS, bionaïve patient group.

Similarly—as in Figure 2—the optimal cut-off value of eight was devised for the IFX: MS bionaïve patient subgroup with a perfect AUC of 1.000 (Figure 4).

**Figure 5:**
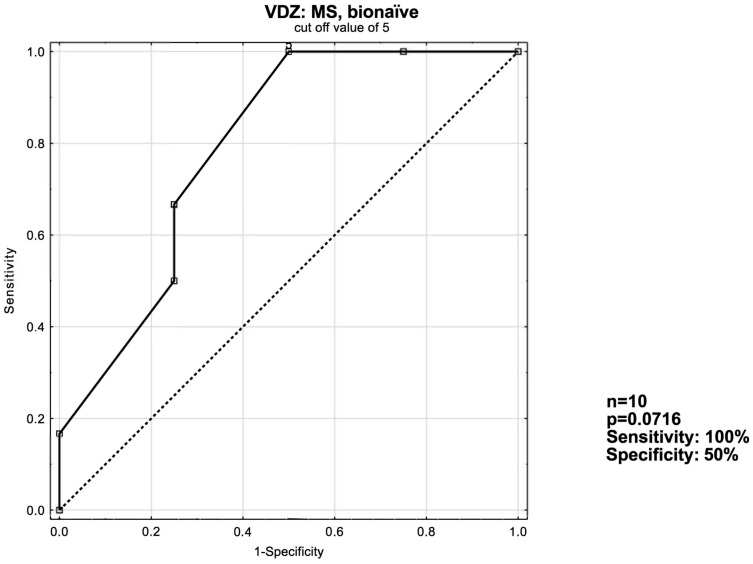
ROC curve for short-term response in the VDZ: MS, bionaïve patient group.

In the VDZ: MS bionaïve patient subgroup, the optimal cut-off value is ≥5 with an AUC of 0.792 (Figure 5).

**Figure 6:**
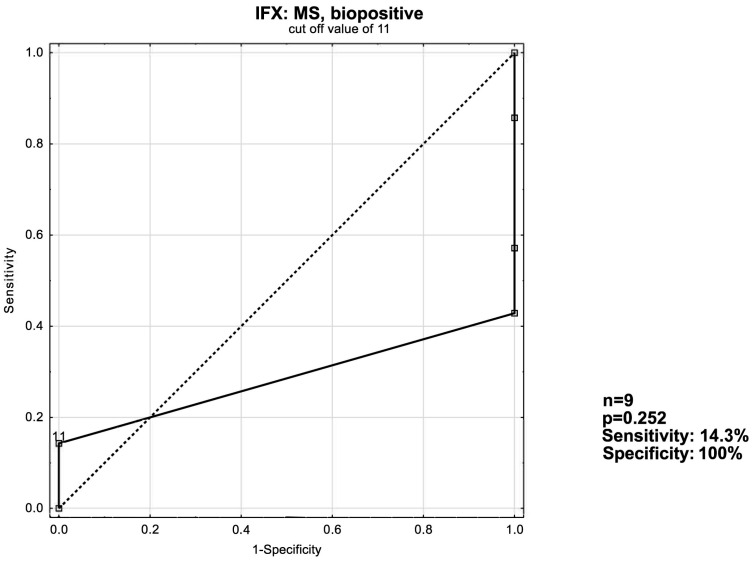
ROC curve for short-term response in the IFX: MS, biopositive patient group.

The IFX: MS biopositive subgroup was the only instance where the trend of higher MS/UCEIS scores coinciding with higher remission induction response rates was in-verted—thus providing the exceptionally low sensitivity value and an AUC of 0.286 (Figure 6).

**Figure 7:**
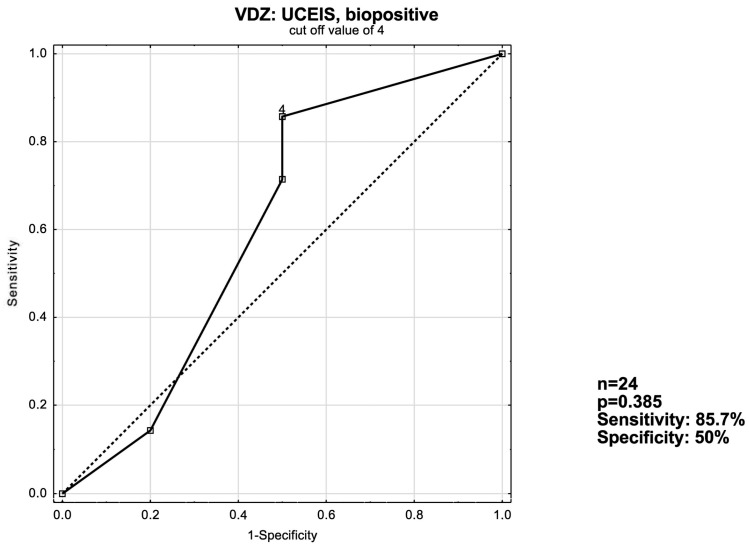
ROC curve for short-term response in the VDZ: UCEIS, biopositive patient group.

For the VDZ: UCEIS biopositive subgroup, a value of ≥4 was the optimal cutoff (Figure 7). This value was paired with an unremarkable AUC of 0.611 and a poor *p*-value of ~0.385.

## 4. Discussion

Inflammatory bowel diseases such as UC pose a heavy economic burden on healthcare systems worldwide [13,14,15,16]; thus, we believe attempts at developing methods of predicting clinical outcomes in such patients are a worthwhile endeavor. In our study, we attempted to measure the predictive value of appropriate UC severity indices’ scores with the hopes of translating initial disease severity assessments into short-term clinical outcome predictions. As UC is a lifelong condition and with patients having to be subjected to indefinite treatment, predicting early outcomes is important as several studies contend that early response to treatment is a predictor of long-term remission [17,18]. Inversely, the lack of early response reduces the long-term efficacy of treatment leading to worse outcomes [19], with one study reporting that roughly 50% of patients without early response to IFX will eventually require colectomy [20].

According to our findings, the MS had a better predictive value than either the UCEIS or the eMS in patients receiving IFX; when the MS is ≥8, 93% of patients will respond to treatment in the short term. In the bionaïve subgroup the response rate reached 100%. Among VDZ recipients, UCEIS proved itself to be superior to the MS; when the UCEIS is ≥4, 70% of patients respond to treatment in the short-term. However, when the bionaïve VDZ patient group was considered separately, the MS proved to be better by a narrow margin; when the MS ≥ 5, 75% of patients respond to treatment vs. 71.4% for UCEIS ≥ 4. What is also noteworthy is that when assessed according to the MS, the non-responder VDZ patient group achieved a statistically significant reduction in their score. This may suggest that the NDP’s criteria may be too harsh on patients receiving VDZ and that they may benefit from being given more time before assessing whether they are responding to treatment so as not to potentially disqualify possible responders prematurely.

Two outliers were presented as follows: in the biopositive IFX patient subgroup, the results were inconclusive with both sensitivity and specificity below acceptable thresholds. In the biopositive VDZ subgroup, when the UCEIS is ≥4, the short-term response rate is 70.6% vs. 68% for MS ≥ 7.

Unfortunately, for all assessed VDZ groups, the *p* values were narrowly above the statistical significance margin.

Among all assessed groups, the eMS proved inferior to both the UCEIS and MS in short-term response prediction.

Several studies analyzed potential methods of predicting outcomes in the treatment of UC; while most assessed outcomes in acute severe colitis [ASC] and for the need for colectomy at initial presentation and subsequent ASC-related hospital admissions [21,22], others focused on factors such as biomarkers [23], nutritional status [24,25], age [26,27], rate of steroid or biological treatment response [28,29] in light of clinical outcomes and disease severity during treatment. Previous research identified anemia and the need for early corticosteroid therapy as predictors for complicated UC [30]. Another concluded that a cut-off value of UCEIS ≥ 6 was appropriate when considering the need for ileal pouch–anal anastomosis creation, especially in the context of prevention of potential malignant transformation [31]. An oral tacrolimus-based study compared UCEIS to eMS in terms of predicting medium- to long-term response and for the need for colectomy concluding that UCEIS is superior to eMS regarding reflecting clinical outcomes and the prediction of medium- to long-term prognosis [32]. Yet, another showed that ASC patients with UCEIS ≥ 7 will require colectomy in 80% of cases, irrespective of applied treatment, and that, in such cases, early colectomy should be considered due to the high risk of non-surgical treatment failure [33]. Overall, there seems to be an overwhelming number of papers that contend UCEIS’s superiority to the eMS [34,35,36,37], with one even stipulating it to be more accurate than MS [38]. Most assert that its advantage stems from a wider range of classification and distinction between depth of ulcers which potentially allows it to detect initial stages of mucosal healing that the endoscopic Mayo Score would otherwise omit. However, we were unable to identify one study which explicitly focused on short-term response to biologics regarding on-admission MS, eMS and UCEIS scores.

Overall, the MS seems to be the most versatile in terms of predicting short-term response in patients receiving IFX; however, such findings may originate from the fact that, while the complete clinical presentation improves (subsidence of extraintestinal manifestations such as peripheral arthritis, anemia, oral sores), the endoscopic presentation does not or does to a less than satisfactory degree—leaving no other option but to change the treatment in the future. While the UCEIS came ahead of the MS in the VDZ study group, this may be because VDZ generally has a longer clinical response time than IFX [39]. As such, extraintestinal manifestations in VDZ recipients may subside later in the treatment when compared with those given IFX. This may suggest that the results presented above could have shifted further in MS’s favor were our study extended to the medium or long term. However, if the luminal response was unsatisfactory, the UCEIS would remain a better predictor due to more precise descriptions of mucosal damage. Furthermore, the results in the VDZ subgroups narrowed above the statistical significance margin or exceeded it greatly. Expansion of study groups and extension of analyzed treatment period may yield stronger results in patients receiving VDZ, especially since—as mentioned above—time to clinical response is significantly longer in VDZ than IFX.

Our study has three main limitations. First, our study was performed in a single center on a relatively homogenous patient group. Second, the data presented was collected and analyzed in a retrospective fashion, making it vulnerable to a degree of subjectivity when compiled for analysis. Third, there was a comparatively modest study group, especially when considering smaller subgroups of analyzed patients.

## 5. Conclusions

The study proved that both the Mayo Score and UCEIS can be considered valid tools for predicting treatment outcomes in the short-term induction treatment period—especially when considering IFX. However, more research should be conducted in this field, especially in terms of study group enlargement and extension of assessed treatment time to the medium or long term.

## Figures and Tables

**Table 1:** Baseline characteristics of the IFX patient sample.

Clinical Remission after 14 Weeks of IFX Therapy				
		Remission	No Remission	*p*-Value
Subjects, *n*		14	5	NA
Sex	women, *n* (%)	6 (42.86%)	2 (40.00%)	0.6641
	men, *n* (%)	8 (57.14%)	3 (60.00%)	
Age, years		36.57 ± 12.35	42.8 ± 15.59	0.3771
	Pancolitis, *n* (%)	9	4	
Extent of lesions	Left colon, *n* (%)	4	0	0.4097
	Rectum, *n* (%)	1	1	
Bionaïve	Yes, *n* (%)	7 (50.00%)	4 (80.00%)	0.2668
	No, *n* (%)	7 (50.00%)	1 (20.00%)	
Glucocorticoid use	Yes, *n* (%)	13 (92.86%)	5 (100%)	0.7368
	No, *n* (%)	1 (7.14%)	0 (0%)	
Disease duration, years		7.57 ± 5.61	8.6 ± 5.77	0.7311
Hemoglobin, g/dL		12.331 ± 2.03	12.53 ± 5.77	0.8664
White blood cell count, ×10^3^/µL		9.76 ± 3.31	10.26 ± 2.51	0.7864
Lymphocyte count, ×10^3^/µL		2.02 ± 1.02	1.69 ± 1.10	0.5875
CRP, mg/L		24.25 ± 39.93	17.10 ± 18.03	0.5335
Calprotectin, µg/L		1213 ± 783.20	814.7 ± 1114.00	0.6098

*p*-value—statistical significance between groups with and without clinical remission after 14 weeks of IFX induction therapy.

**Table 2:** Baseline characteristics of the VDZ patient sample.

Clinical Remission after 14 Weeks of VDZ Therapy				
		Remission	No Remission	*p*-Value
Subjects, *n*		20	14	NA
Sex	women, *n* (%)	11 (55.00%)	4 (28.57%)	0.1194
	men, *n* (%)	9 (45.00%)	10 (71.43%)	
Age, years		36.12 ± 12.10	38.29 ± 16.11	0.9860
	Pancolitis, *n* (%)	11 (55.00%)	8 (57.14%)	
Extent of lesions	Left colon, *n* (%)	8 (40.00%)	5 (35.71%)	1.000
	Rectum, *n* (%)	1 (5.00%)	1 (7.14%)	
Bionaïve	Yes, *n* (%)	14 (70.00%)	4 (28.57%)	**0.0204**
	No, *n* (%)	6 (30.00%)	10 (71.43%)	
Glucocorticoid use	Yes, *n* (%)	19 (95.00%)	12 (85.71%)	0.3650
	No, *n* (%)	1 (5.00%)	2 (14.29%)	
Disease duration, years		7.95 ± 5.72	7.714 ± 6.64	0.9125
Hemoglobin, g/dL		13.57 ± 1.36	13.86 ± 1.59	0.5785
White blood cell count, ×10^3^/µL		8.54 ± 3.06	8.86 ± 3.07	1.0000
Lymphocyte count, ×10^3^/µL		1.96 ± 0.91	2.24 ± 1.38	1.0000
CRP, mg/L		10.11 ± 9.87	12.67 ± 25.85	0.6358
Calprotectin, µg/L		1385.1 ± 702.37	752.00 ± 527.6	0.0758

*p*-value—statistical significance between groups with and without clinical remission after 14 weeks of VDZ induction therapy. Statistically significant values were outlined in bold.

**Table 3:** Mean UCEIS, MS and eMS scores in IFX patients.

		Before Treatment	After 14 Weeks of Treatment	*n*	*p*-Value
UCEIS	Induction	4.786 ± 1.528	2.500 ± 1.160	14	**<0.001**
	No induction	4.800 ± 1.483	4.400 ± 1.342	5	1.0
	Total	4.790 ± 1.475	3.000 ± 1.453	19	**0.0012**
eMS	Induction	2.571 ± 0.646	1.500 ± 0.519	14	**<0.001**
	No induction	2.800 ± 0.447	2.600 ± 0.548	5	N/A
	Total	2.632 ± 0.597	1.790 ± 0.713	19	**<0.001**
MS	Induction	8.929 ± 1.492	3.2857 ± 1.326	14	**<0.001**
	No induction	6.600 ± 2.302	6.600 ± 2.510	5	0.371
	Total	8.316 ± 1.976	4.158 ± 2.218	19	**<0.001**

*p*-value—statistical significance between groups before treatment and after the 14th week treatment mark. Statistically significant values were outlined in bold.

**Table 4:** Mean UCEIS, MS and eMS scores in VDZ patients.

		Before Treatment	After 14 Weeks of Treatment	*n*	*p*-Value
UCEIS	Induction	4.550 ± 1.146	2.200 ± 1.7652	20	**<0.001**
	No induction	3.786 ± 1.528	3.500 ± 1.829	14	0.75183
	Total	4.235 ± 1.350	2.735 ± 1.879	34	**0.00208**
eMS	Induction	2.800 ± 0.410	1.500 ± 1.051	20	**<0.001**
	No induction	2.571 ± 0.646	1.928 ± 1.071	14	0.0771
	Total	2.706 ± 0.524	1.677 ± 1.0652	34	**<0.001**
MS	Induction	7.55 ± 2.013	2.600 ± 2.036	20	**<0.001**
	No induction	6.428 ± 2.441	5.071 ± 2.200	14	**0.005546**
	Total	7.062 ± 2.299	3.618 ± 2.412	34	**<0.001**

*p*-value—statistical significance between groups before treatment and after the 14th week treatment mark. Statistically significant values were outlined in bold.

## Data Availability

The data supporting this study’s findings is available from the corresponding author (RF) upon reasonable request.
